# Suffering of Common Mental Disorders but Still at Work: A Longitudinal Study During Periods of Differences in Regulations for Having Sick Leave

**DOI:** 10.1007/s10926-025-10269-4

**Published:** 2025-01-31

**Authors:** Magnus Helgesson, Klas Gustafsson, Constanze Leineweber

**Affiliations:** 1 Division of Insurance Medicine, Department of Clinical Neuroscience, Karolinska Institutet, SE-17177 Stockholm, Sweden; 2 Department of Public Health and Caring Sciences, Public Health, Working Life and Rehabilitation, Uppsala University, SE-75122 Uppsala, Sweden; 3 Department of Psychology, Stockholm University, SE-10681 Stockholm, Sweden

**Keywords:** Common mental disorder, Sick leave, Working conditions, Flexible work, Telework, Job control

## Abstract

**Purpose:**

The aim was to analyse the relationships between work environment characteristics and periods of sick leave (> 14 days) in individuals diagnosed with common mental disorders (CMDs) across 1993–2014. Additionally, the study describes changes in the work environment and sick leave trends over these two periods.

**Methods:**

From the Swedish Work Environment Surveys (SWES) 1993–2013, participants with a diagnosis of a CMD up to five years before the interview were drawn (*n* = 3795). Risk ratios (RRs) of the annual average number of sick leave days one year after the interview in SWES (1993–2014) were calculated for various work environment characteristics, along with 95% confidence intervals (CIs).

**Results:**

Having flexible working hours (RR 0.65: 0.46–0.91) and having an active job (RR 0.60: 0.41–0.88), that is, high job demands and high job control, were associated with a significantly decreased risk for a spell of sick leave > 14 days after adjusting for interview year, age, sex, and educational level. There was a tendency that also some aspects of job control, i.e. influence over working hours (RR 0.79: 0.62–1.01) and influence over work pace (0.80: 0.62–1.03), were associated with a lower risk of having  > 14 days of sick leave. There was a more substantial effect on these work environment factors in 2009–2013 than 1993–2007.

**Conclusion:**

Enabling flexible working hours and influencing work pace and working hours can decrease the risk of sick leave among employees diagnosed with a CMD.

**Supplementary Information:**

The online version contains supplementary material available at 10.1007/s10926-025-10269-4.

## Introduction

The incidence, or more accurately, diagnostics of common mental disorders (CMDs), that is, depression, anxiety, and stress-related disorders, has increased significantly during the 2000s in Sweden [1–3]. People with CMDs are one of Sweden's most prominent groups on sick leave and CMDs are a steadily increasing reason for having sick leave, especially among young women. Foremost, diagnoses of stress-related disorders have risen sharply since 2010 [4]. Simultaneously, the rate of sick leave due to these disorders has increased much [3]. The inability to work due to CMD has turned out to be very costly for society and employers and also individuals with CMDs, as they risk ending up outside the labour market [5]. However, studies show that many individuals with CMD can and do work despite their illness [1]. Therefore, it is essential to elucidate which work characteristics relate to successful work participation and what characterises successful work participation for employees with CMDs to preserve welfare.

Several factors have been seen to affect sick leave among individuals suffering from CMDs, including individual characteristics such as sex, age, and educational level [1]. Many researchers have also shown that a good work environment provides better chances for staying at work also when having a CMD [6]. On the other hand, a poor psychosocial work environment, characterised by a combination of high job demands and a low degree of job control and social support, may lead to spells of sick leave [7–12]. In addition, work organisational factors, such as the possibility to adapt the work situation to one’s daily level of functioning by, for example, working from home and having influence over one’s working hours, can affect the person's work ability [13].

Regulations significantly impact whether an individual can be granted benefits from sickness insurance when being disabled or sick. Many countries have a strict set of criteria that must be met to receive sick leave benefits. Other countries, such as Sweden, have a more liberal system. However, since its foundation in 1955, the Swedish Insurance Agency has had many changes in the regulations for sickness insurance [14]. However, most were less ground-breaking than the changes in 2008 when the possibility of receiving a disability pension was reserved only for persons with life-long incapacitation of their workability. Before the reforms in 2008, time-restricted disability pension was very common. At the same time, the sick leave benefits became time-restricted, which led to many being denied the right to have sick leave benefits without the possibility of applying for disability pension. This has supposedly led to an increase in people suffering from ill health who now have to work to earn their living. We hypothesise that beneficial work factors become increasingly crucial for maintaining the ability to work when the workforce includes an increasing number of persons close to being outside work due to illness. The possibility of assessing the periods before and after the changes in 2008 is an opportunity to study the association of psychosocial and organisational work factors and sick leave in periods with low and high access to welfare benefits.

The current study elaborated on prognostic factors associated with having sick leave in a population of individuals with a CMD. The study had two main objectives: first, to analyse the relationship between organisational and psychosocial work environment and individually compensated sick leave 1993–2014, both generally and over the periods 1993–2008 and 2009–2014, for individuals with CMDs; and second, to describe changes in the exposure of psychosocial and organisational work environment for individuals with CMD during these periods.

## Methods

### Study Population

Participants were drawn from the biennial Swedish Work Environment Surveys (SWES) 1993–2013 (*n* = 100,719). Between 1993 and 2013, the annual response varied between 82 and 66% [15]. For more details on SWES, see e.g. Helgesson et al. [13]. In this study, only those who were diagnosed with a common mental disorder (CMD) (diagnose codes F32 to F43 in International Classification of Disorders – version 10 (ICD 10) or codes 296, 298, 300, 301, 308, and 311 from ICD-9) or received treatment with antidepressants (Anatomical Therapeutic Chemical code (ATC): N06A) at the year or up to five years before they answered the SWES were included (*n* = 3795).

### Data Sources

Information on sociodemographic data, including sex, age, and educational level, was derived from the Longitudinal Integrated Database for Health Insurance and Labour Market Studies (LISA) [16]. Information on sick leave was derived from the Midas database, hosted by the Swedish Social Insurance Agency (SSIA), and high-quality health data were gathered from the National Patient Register [16]. These registers were linked to the survey data in SWES by the unique national identification number.

### Measurements

#### Outcome Variable

Periods of sick leave, irrespective of cause, were obtained from registers. Regulations for receiving public sick leave benefits changed profoundly in 2008. The first day of a sick leave period was not compensated during the study period, and days 2 to 14 were compensated by the employer (except for 15 months from 1st of January 1997 to 31st of March 1998 when days 2 to 28 were compensated by the employer) [14]. Thus, in this study, for the vast majority of the study period, a spell of a sick leave period of 14 days or more was compensated by SSIA and thus was available in registers. Sick leave was defined as any day of compensated sick leave, mainly meaning more than 14 days from SSIA during the year after participation in SWES. To have proximity between the measurements of the work environment, which is conducted from November and ending in March, and sick leave, sick leave was measured from the 1st of April in the year of SWES until the 31st of March in the following year. For example, for participants in SWES 1993, sick leave was indicated if there were any compensated sick leaves between the 1st of April in 1993 and the 31st of March in 1994. Two days of half-time and four days of quarter-time sick leave were counted as one net day of sick leave.

#### Exposure Variables

Twelve psychosocial and organisational work factors were chosen [13] to measure exposures in the work environment. All items in the SWES were self-reported and had good validity [17, 18].

Flexible working hours were measured by one item, i.e. “In general, are you able to determine your working hours within certain limits?” with response options “Yes, I have flexible hours (begin and end work not at exact times, but within certain specified periods)” and “Yes, I have relatively free working hours in a different way” as having flexibility and “No, I am generally unable to affect my working hours” coded as not having flexibility.

Possibilities to work from home were measured by the item “How much of your normal working time do you usually work at home?”. Having this possibility at least some hours a week indicated the possibility to work from home, while “Don’t work from home” was coded as no.

Social support at work was measured by two single items (“Are you able to get support and encouragement from supervisors, when your work feels difficult?” and “Are you able to get support and encouragement from colleagues when work feels difficult?”). Both were answered on a 4-point Likert scale from 1 = always to 4 = never. If social support from either supervisors or colleagues was received always or most of the time, social support was indicated (coded as '1’). Otherwise, social support was coded as absent (‘0’).

One item was used to measure an open atmosphere at work (“Are you reluctant to express critical views in the workplace regarding your working conditions?”). Responses were given on a four-point Likert scale reaching from 1 = always to 4 = never. Being not hesitant (never and mostly not) was coded as ‘1’.

Four items indicating high job demands were chosen in line with previous research [13, 19]. These were (a) “Do you have so much work that you must miss lunch, work late, or take work home?” (1 = *Not at all/rarely in the last three months*), (b) “Is your work so stressful that you do not have time to talk or even think about something other than work?” (1 = *No, not at all* or* about 1/10 of the time*), (c) “Does your work require your full attention and concentration?” (1 = *About 1/10 of the time or no, not at all*), and (d) “Have far too much or far too little to do at work” (1 = *Neither nor, that is not too much or too little*). Another four items indicated *job control* at work are (e) “Are you able to determine when various working duties are to be carried out (for example, by choosing to work a bit faster on some days and taking it easier other days)?” (1 = *Always* or *Mostly*), (f) “Do you participate in decisions on the arrangement of your work (for example, what is to be done, how to do it or who will work with you)?” (1 = *Always* or *Mostly*), (g) “Do you have the opportunity to determine your work pace?” (1 = *Nearly all the time* or *about ¾ of the time*), or (h) “have too little (much) influence at work” (1 = *Always* or *Mostly* (1 = *Neither nor*)). Item (a) was answered on a five-point scale reaching from 1 = every day to 5 = not at all/seldom the past three months. Items (b), (c), and (g) were answered on a five-point scale, reaching from 1 = Nearly all of the time to 5 = No, not at all. Item (d) was answered on a five-point scale, reaching from 1 = far too much to do to 5 = far too little to do. In similarity, item (h) was answered from 1 = too little influence to 5 = too much influence. Items (e) and (f) were answered on a four-point scale, reaching from 1 = all the time to 4 = no, not at all. Cut-offs were chosen to indicate a beneficial work environment and are given in parentheses after the items.

A measure to indicate an active job, the most beneficial according to theory, was calculated following Magnusson-Hansson et al. [19]. If a person answered positively to at least two indicators of job demands or control, this indicated high job demands and high job control, respectively. Those who indicated having high job demands and high control were coded as having an active job; all remaining possibilities were coded as having less beneficial job conditions.

### Confounders

Year of interview, age, sex, and educational level were considered potential confounding variables. All these factors were derived from registers. The age was calculated as the year of the interview minus the year of birth. Sex was binary (male or female). Educational level was based on the Swedish classification system of education (SUN2000) and coded into three groups (elementary school, upper secondary school, or university).

### Statistical Methods

The results were organised into an analytic part and a descriptive part. In the first part, risk ratios (RRs) of the annual average of sick leave days the year after the SWES interview were calculated, pooled, and divided into two periods: 1993–2007 and 2009–2013. Corresponding data regarding sick leave were measured for the following SWES: April 1994 to April 2008 and April 2010 to April 2014. The “Proc Genmod” procedure in SAS computed relative risks between work environment factors and subsequent sick leave among persons suffering from CMD. The first step calculated a model controlled only for the interview year. In the next step, age, sex, and educational level were also controlled for. All data management and calculations were performed in SAS 9.4. The analyses were stratified by period, that is, the periods 1993–2007 and 2009–2013 (and sick leave between 1993–2008 and 2009–2014). The number of participants in each period was somewhat different as data regarding prescribed antidepressant medication were available only from 2005.

The second part illustrates how the proportions of individuals with different work environment exposures changed between 1993 and 2013. The descriptive part was calculated and presented without adjustment for any confounders.

## Results

Of the 3795 persons with CMD included in this study, 2596 (68.4%) were female, and 1199 (31.6%) were male (Table 1). The mean age was 46.1 (Std = 11.1), and the majority (55.3%) were over the age of 46 years. Most participants had a secondary school education (n = 1783; 49.1%) or a university degree (*n* = 1294; 35.6%), and fewer had an elementary school education only (*n* = 556; 15.3%). Most individuals in the population were included by having prescribed antidepressant medication (*n* = 2558; 67.4%).

**Table 1:** Distribution of the study group by age, sex, educational level, and source of diagnosis for common mental disorders (CMD) (*n* = 3795)

	Study group	
Variables	*N*	%
Age		
16–25	172	4.5
26–35	568	15.0
36–45	957	25.2
46–55	1190	31.4
56–64	908	23.9
Sex		
Woman	2596	68.4
Men	1199	31.6
Educational level		
Low (Elementary school)	556	14.7
Medium (upper secondary school)	1783	47.0
High (University)	1294	34.1
Source of diagnosis for CMD		
Hospital diagnosis	647	17.1
Prescribed medicine	2558	67.4
Both	590	15.6
Total	3795	100

Table 2 shows the risk ratios for having a spell of more than 14 days of sick leave the following year after participation in the work environment survey by the organisational and psychosocial work environment. Model 1, only adjusted for the interview year in SWES, indicates a statistically significantly reduced risk for sick leave of 14 days or more when having flexible work hours, possibilities to work from home, and influence over work time and pace. After adjusting for age, sex, and educational level, only having flexible working time was still associated with a decreased risk for 14 days or more of sick leave (RR 0.73: 0.57–0.93). There was also a clear tendency that working from home (RR 0.79: 0.58–1.08), having influence over working hours (RR 0.79: 0.62–1.01), and influence over work pace (0.80: 0.62–1.03) were associated with a decreased risk of having a spell of sick leave over 14 days.

**Table 2:** The risk ratios (RRs) with 95% confidence intervals (CI) for 14 days of compensated sick leave (1993–2014) among individuals diagnosed with common mental disorders (CMD) for various work environment variables (n = 3795)

			Model 1^c^				Model 2^d^	
Exposure variables	N^a^	n^b^	RR	*p*-value	RR	CI		*p*-value
Flexible working hours								
No	1520	124	**1**		**1**			
Yes	2179	124	**0.70**	**0.00**	**0.73**	**0.57—0.93**		**0.01**
Working from home								
No	2814	202	**1**		1			
Yes	897	46	**0.73**	**0.05**	0.79	0.58—1.08		0.14
An open atmosphere at work								
No	2649	178	1		1			
Yes	935	55	0.88	0.39	0.90	0.67—1.21		0.48
Overtime								
No	2220	153	1		1			
Yes	1521	103	0.97	0.78	0.96	0.75—1.22		0.72
Time to talk								
No	2374	167	1		1			
Yes	1385	89	0.90	0.39	0.93	0.72—1.20		0.57
Need to concentrate								
No	3547	240	1		1			
Yes	203	17	1.20	0.45	1.16	0.71—1.92		0.55
Just enough to do								
No	2196	156	1		1			
Yes	1563	102	0.92	0.47	0.98	0.77- 1.25		0.86
Influence over working hours								
No	1873	147	1		1			
Yes	1839	105	**0.72**	**0.01**	0.79	0.62—1.01		0.06
Influence over how to work								
No	1138	81	**1**		1			
Yes	2594	171	0.94	0.63	0.94	0.72—1.21		0.62
Influence over work pace								
No	2204	166	1		1			
Yes	1508	88	**0.76**	**0.03**	0.80	0.62—1.03		0.08
Influence at work								
No	1611	116	**1**		1			
Yes	2141	139	0.91	0.45	0.93	0.73—1.18		0.55
Active job^e^								
No	2372	171	1		1			
Yes	1422	88	0.87	0.26	0.88	0.68—1.13		0.30
Social support								
No	1606	116	1		1			
Yes	2189	143	1.09	0.47	1.10	0.87—1.40		0.43

^a^Number (N) of the exposure categories

^b^ Number of cases (n)

^c^Model 1: Risk ratio (RR) adjusted for the year of the interview, presented without showing confidence intervals

^d^Model 2: Risk ratio (RR) adjusted for year of interview, age, sex, and educational level

^e^ High job demand and high job control

Significant figures are shown in bold (p < 0.05)

Table 3 provides the risk ratios for having a spell of 14 days or more of sick leave in 1993–2008 or 2009–2014. A flexible working time (RR 0.65: 0.46–0.91) and an active job (RR 0.60:0.41-0.88) were associated with a decreased risk for more than 14 days of sick leave in 2009–2014. During the latter period, influencing how to work (RR 0.78: 0.55–1.12) tended to be most important for having a decreased risk of a spell of sick leave of more than 14 days. Interaction analyses between periods and the work factors showed significant differences between the periods regarding the factors of active jobs (*p* = 0.01) and just enough to do (*p* = 0.04).

**Table 3:** The risk ratios (RRs) with 95% confidence intervals (CIs) for sickness absence of 14 days or more in the two time periods, i.e. 1993–2008 and 2009–2014, among workers diagnosed with common mental disorders (CMD) for various work environment variables (n = 3795)

	1993–2008						2009–2014						P-value
Exposure variables	N^a^	n^b,,^	RR^c^	RR^d^	CI		N^a^	n^b^	RR^c^	RR^d^	CI		Interaction^e^
Flexible working hours													
No	610	55	1	1			910	69	1	1			
Yes	847	62	0.82	0.85	0.60	1.20	1332	62	**0.62**	**0.65**	**0.46**	**0.91**	0.28
Working from home													
No	1149	98	1	1			1665	104	1	1			
Yes	312	19	0.72	0.77	0.48	1.24	585	27	0.73	0.80	0.53	1.22	0.88
An open atmosphere at work													
No	1017	80	1	1			1632	98	1	1			
Yes	331	23	0.88	0.94	0.60	1.47	604	32	0.89	0.88	0.59	1.32	0.58
Overtime													
No	858	75	1	1			1362	78	1	1			
Yes	649	51	0.89	0.89	0.63	1.25	872	52	1.04	1.03	0.73	1.45	0.56
Time to talk													
No	930	86	1	1			1444	81	1	1			
Yes	585	40	0.73	0.78	0.54	1.12	800	49	1.10	1.12	0.79	1.60	0.19
Need to concentrate													
No	1425	118	1	1			2122	122	1	1			
Yes	88	8	1.06	1.01	0.48	2.09	115	9	1.39	1.34	0.68	2.67	0.66
Just enough to do													
No	890	85	1	1			1306	71	1	1			
Yes	630	42	0.70	0.74	0.52	1.06	933	60	1.20	1.32	0.93	1.84	**0.04**
Influence over working hours													
No	760	70	1	1			1113	77	1	1			
Yes	709	50	0.75	0.81	0.57	1.16	1130	55	**0.70**	0.77	0.54	1.08	0.80
Influence over how to work													
No	480	37	1	1			658	44	1	1			
Yes	1027	87	1.10	1.12	0.77	1.64	1567	84	0.79	0.78	0.55	1.12	0.20
Influence over work pace													
No	850	81	1	1			1354	85	1	1			
Yes	616	41	**0.68**	0.71	0.49	1.03	892	47	0.84	0.87	0.61	1.24	0.56
Influence at work													
No	677	57	1	1			934	59	1	1			
Yes	845	69	0.98	1.02	0.72	1.43	1296	70	0.86	0.86	0.61	1.20	0.46
Active job													
No	976	76	1	1			1396	95					
Yes	557	51	1.17	1.22	0.87	1.72	865	37	**0.62**	**0.60**	**0.41**	**0.88**	**0.01**
Social support													
No	668	60	1	1			938	56	1	1			
Yes	865	67	0.88	0.87	0.62	1.22	1324	76	0.96	0.95	0.68	1.34	0.65

^a^Number (N) of the exposure categories

^b^Number of cases (n)

^c^Risk ratio (RR) for sickness absence, only adjusted for the interview year, presented without showing confidence intervals

^d^Risk ratio (RR) for sickness absence with 95% confidence interval (CI), adjusted for year of interview, age, sex, and educational level

^e^Value for interaction between period and the work factor

Significant figures are shown in bold (p < 0.05)

There was a slight increase in exposure to a beneficiary work environment, foremost by those having social support, flexible working hours, working from home (telework), and a good atmosphere at work (possibilities to utter critical points at work) (Supplementary Fig. 1). Relatively stable curves could be noticed for different measures of influence over work, i.e. influence over work time/hours, how to work, and general influence at work. Also, having enough to do and having an active job were relatively stable work characteristics. However, the figures indicated a general decrease in the proportion influencing work pace and job demands measured as having *no* overtime, having time to talk in the workplace, and having *no* need to concentrate.

## Discussion

### Main Findings

Only flexible working hours were associated with a lower risk of having sick leave for the whole period; both flexible working hours and an active job were significantly associated with a decreased risk of having a sick leave spell over 14 days in the period after the significant change in the rules in sickness insurance. There was also a tendency that influence over working hours and work pace were associated with a decreased risk of a spell of sick leave over 14 days. Exposure to many dimensions of job control and social support had a positive trend in 2009–2014, while job demands and influencing over work pace worsened.

### Changes in Regulations (2008)

In Sweden, levels of sick leave have varied noticeably over the years and were often related to simultaneous changes in regulations within the Swedish social insurance system [14]. The regulations have, however, not frequently changed as comprehensively as in 2008. After this change, the granting rate of disability pension was about one-tenth compared to before 2008 [20]. This regulation change entailed a workforce from 2008 onwards where a higher proportion previously could either have sick leave or be granted disability pension.

Spells of sick leave pose challenges to employees and employers, impacting productivity and well-being. In contexts with strict sickness benefit regulations, preventive measures become paramount. Employees may hesitate to take time off due to financial concerns or job insecurity, leading to increased presenteeism and potentially a higher severity of illness in the long run [21]. Employers may be reluctant to hire new staff due to the high risk of productivity losses [22]. This may start a downward spiral in terms of health status and productivity in the workplace.

This study shows noticeable differences between the two periods among those diagnosed with a CMD and having an active job and those with influence over work time, which involves different forms of control and flexibility over work conditions. Over the last decades, studies have found an increase in sick leave related to CMDs, and it may be linked to a simultaneous rise in harmful psychosocial exposure in many occupations [12, 23–25]. Job demands have increased during the last two decades, requiring a concurrent increase in job control and social support. A balance between job demands and resources is needed to maintain a sustainable workforce [23, 26].

### Flexible Work Time

Flexible work time encompasses arrangements that allow employees to vary their work hours, offering some influence over their schedules. Research suggests flexible work time contributes to higher employee satisfaction, work–life balance, and overall well-being [27, 28]. By accommodating individual needs and preferences, flexible work time can mitigate stressors associated with rigid schedules and facilitate better management of personal and professional responsibilities [29]. Flexible work time can, therefore, serve as a preventive measure against sick leave by addressing various factors contributing to ill health [29]. For instance, it enables employees to attend medical appointments without disrupting their workday, facilitating timely healthcare access and preventive screenings. Moreover, flexible schedules accommodate individual chronotypes and preferences, reducing fatigue and enhancing sleep quality, which is linked to resilience against illness [30]. If employers prioritise supportive policies and practices to leverage the benefits of flexible work time, better health and high productivity may be maintained [29, 30]. Flexible work time offers a promising strategy for preventing sick leave in environments with strict benefit regulations. By empowering employees to manage their schedules and prioritise health needs, organisations can mitigate the impact of illness on productivity and well-being. The share of individuals with CMDs having flexible working hours was higher in the last period compared to the first period. However, there has been a declining trend from 2009 to 2013. The COVID-19 pandemic has changed this, and hopefully, there will be a change in this trend. Individuals with CMD may benefit from working from home [31].

### Active Jobs

Active jobs, characterised by high job demands and job control, mitigate sick leave rates among individuals with CMDs. In theory, high job demands entail a challenging workload, while high job control grants employees autonomy and decision-making authority over their tasks. Some research suggests that when these two factors coexist, they create a dynamic work environment that fosters engagement, motivation, and resilience, as well as lower levels of stress and burnout as they perceive challenges as manageable and within their control [6, 32]. Active jobs may buffer against sick leave during optimal conditions by promoting employee well-being and resilience. However, active jobs have been described as problematic, especially among women, where job control is insufficient to balance the high demands, for example, in healthcare work [12, 33–35]. The share of active jobs has been relatively stable for the whole period, from 1993 to 2013, with around 40 per cent. The element of job control may be essential within this category. Job demands have increased in most occupations since the 1990s. High job control may mitigate the detrimental effects of these high demands, manifested in the lower risk of having sick leave over 14 days.

### Job Control

Job control, measured by influence over working hours and pace, tends to protect against long periods of sick leave. A Norwegian study has shown that control over work pace was especially protective for sick leave in a population of home care workers [36]. In an Australian study, individuals with any disability were shown to have a 24% higher risk of having sick leave compared to disabled individuals with high job control [37]. It is of higher importance for a group of individuals with any disabling disorder to have a favourable work environment regarding job control to have the possibility to stay at work, as it allows them to adapt their work to their daily abilities. Having high job control can thus be the key for those having a disabling CMD to have the opportunity to stay at work. The share of working individuals with high job control seems stable, with a slight positive development, from 40 per cent to around 60 per cent depending on a specific question. If this share were increased, a higher proportion of individuals with CMDs could remain in the workforce, benefiting society, employers, and, not least, themselves.

### Sex and Educational Level

Our analysis shows that educational level and sex influence the association between work environment factors and later sickness absence. In some analyses, the association diminishes with the adjustment of educational level, age, and sex. In Sweden, there have been considerable differences between men and women regarding the receipt of sick leave benefits; in total, women account for two-thirds of the utilisation of health insurance [34]. Sweden also has a highly gender-segregated labour market, where women-dominated branches such as healthcare and caring for older people, that is, jobs with low work (time) control, have a high proportion of individuals on sick leave [38]. Higher education has long been seen as a way to receive better job opportunities and improve work conditions. Individuals with a higher educational level are also more likely to negotiate favourable work conditions, including flexible hours, telework, and access to professional development opportunities. Technological skills are also increasingly valued in the workplace due to the rapid development of digital tools [39].

### Strengths and Limitations

The major strength was the prospective design, where we obtained sick leave spells from high-quality registers for one year after the interview, minimising the risk of recall bias. Moreover, the number of interviews was based on representative samples with a reasonable response rate and where the items have acceptable reliability and validity [17, 18].

Limitations of the present study were the long study period, meaning there may have been substantial changes and variations in covariates and diagnostics of CMDs during the study period. Information on prescribed drugs was available only from 2005, which reduced the inclusion of participants in the study from 1993 to 2003. Due to the few participants, we could not perform subgroup analyses regarding those included by diagnosis from healthcare or those included by prescribed drugs. Moreover, the generalisability of the result may be limited to a working population with a CMD, as those in work often have better general health compared to a general population with CMDs. A selective population with few participants in some strata made stratification of factors such as sex, age, and educational level impossible. Finally, the registers had shortcomings; only those diagnosed with a CMD in hospital care were included. Those diagnosed in primary care with prescribed antidepressants were included in the Prescribed Drug Register. Unfortunately, however, medicine prescriptions were not registered until 2005. Another limitation of this study is the potential risk of significant findings arising by chance, as we consider thirteen different factors from the work environment. We considered applying Bonferroni correction to mitigate this risk, but as we have only a very small number of significant findings, this seemed superfluous. Still, we acknowledge that despite these precautions, the possibility of chance findings cannot be completely ruled out, and we are consequently quite cautious in our interpretation. At the same time, many estimates are close to significance.

## Conclusions

Having flexible work time and having an active job were associated with a decreased risk for a spell of sick leave over 14 days among employees with CMDs in a period with stricter rules within the sickness insurance. It is also evident that exposure to high job control had a positive trend in 2009–2013, while job demands increased and the influence over work pace decreased during the same time. This may be problematic from the perspective of sustainability.

By creating a supportive work environment that values employee well-being and autonomy, organisations can empower individuals to manage their health proactively and minimise the impact of illness on productivity, especially in settings where stricter rules for having sickness benefits occur. Maintaining a healthy workforce is paramount for employees and employers in the face of more strict regulations surrounding sickness benefits.

## Supplementary Information

Below is the link to the electronic supplementary material.

Supplementary file1 (DOCX 223 KB)

## Data Availability

The data for this study are available from Statistics Sweden, the Swedish Social Insurance Agency, and the Swedish National Board of Health and Welfare. However, the data are not publicly available and can be used only with ethical permission.
