# Supplementary material for: Suffering of Common Mental Disorders but Still at Work: A Longitudinal Study During Periods of Differences in Regulations for Having Sick Leave
Source: J Occup Rehabil. 2025 Jan 31;36(2):373–82. doi: 10.1007/s10926-025-10269-4 (PMC13099786; doi:10.1007/s10926-025-10269-4)
Supplement: Supplementary file 1 — Supplementary file1 (DOCX 223 KB) [file 10926_2025_10269_MOESM1_ESM.docx]

**Supplementary figures 1a-n.** The development of the different work environmental characteristics and sick leave (%) over the study period, i.e., 1993-2007 and 2009-2013.
